# From dual inhibition to precision selectivity: the molecular rationale and clinical evolution of next-generation PARP1-selective inhibitors in solid tumors

**DOI:** 10.3389/fonc.2026.1894818

**Published:** 2026-07-24

**Authors:** Maha M. Ayoub, Shayma A. Osman, Reem M. Alkarbi, Rafia Anjum, Ahmed Malki

**Affiliations:** Department of Biomedical Sciences, College of Health Sciences, Qatar University, Doha, Qatar

**Keywords:** BRCA mutation, DNA damage response, hematologic toxicity, homologous recombinant deficient solid tumors, PARP inhibitors, PARP1 selectivity, synthetic lethality

## Abstract

Poly (ADP-ribose) polymerase inhibitors (PARPi) have revolutionized Homologous recombination deficient (HRD) solid tumors by using the synthetic lethality which BRCA 1/2 mutated cancer cells depend on for DNA repair. Standard PARP inhibitors such as olaparib, rucaparib, niraparib, and talazoparib have been shown to inhibit both PARP1 and PARP2, and have shown significant clinical efficacy in breast, ovarian, prostate, and pancreatic cancers. However, their clinical efficacy is limited by their hematological toxicities such as anemia, thrombocytopenia, and neutropenia that requires reduction in dose and treatment discontinuation which compromises their therapeutic efficacy and long-term use. This review focuses on molecular mechanism, preclinical and clinical evidence highlighting the shift from standard dual PARP 1/2 inhibitors into selective PARP1 inhibitors. Molecular and preclinical evidence has shown that inhibition of PARP1 is sufficient to induce synthetic lethality in HRD tumors, while inhibition of PARP2 causes hematopoietic stem and progenitor cell (HSPC) toxicity via impairment of erythropoiesis. These limitations led to the use of next-generation selective PARP1 inhibitors, such as saruparib (AZD5305), with approximately 500-fold higher selectivity for PARP1 over PARP2 with potent antitumor activity and reduced hematological toxicity. The clinical efficacy and tolerability of selective PARP1 inhibitors provides a significant opportunity to use them as combination therapy which was previously limited due to combined myelosuppression. Collectively PARP1 inhibitors have demonstrated clinically impactful advancement that exhibits anticancer effect without causing significant hematological toxicity. They have potential of treating patients who benefit from PARP1 inhibitor-based precision oncology.

## 1 Introduction

Cancer remains one of the major concerns for the public health sector around the world. In a 2020 study, researchers estimated that more than 19 million new cases of cancer were reported globally, and around 10 million people died from the disease in the same year (1). Most cancer-related deaths are associated with metastatic disease. In metastatic cancer, tumor cells migrate from the primary tissue site to distant organs, spreading the disease to other parts of the body (2). This process typically occurs in the later stages of the disease and worsens the prognosis. Enabling this malignant progression is the hallmark of genomic instability at molecular level. Tumor suppressor genes such as BRCA1 and BRCA2 play a critical role in maintaining this genomic instability by repairing damaged DNA. Mutations in these genes can cause accelerated accumulation of mutations (3).

An intricate DNA repair system counteracts different forms of DNA lesions to maintain genomic integrity and this mechanism is known as the DNA damage response (DDR) pathway. DDR pathways are classified into three interconnected parts: sensor to detect DNA damage, signal transducer to trigger signaling cascades, and effector that initiates DNA repair. The genome experiences subtle change due to environmental insults such as radiation or generated endogenously generating reactive oxygen species (ROS) (4). These subtle changes in DNA such as single-strand breaks (SSBs) are repaired through base excision repair (BER) pathway. Damaged bases are excised and replaced with newly synthesized DNA. Then the AP site is cleaved to form 3′ OH terminus by the apurinic/apyrimidinic (AP)‐endonuclease (APE) at the damage site. Finally, the nucleotide gap produced by the lesion base removal is sealed by the DNA polymerase and DNA ligase (5).

The key DDR enzymes, PARP1 and PARP2, sense DNA damage and repair single stand-breaks (SSBs) through the Base Excision Repair (BER) pathway (6). Poly (ADP-ribose) polymerase inhibitors (PARPi) induce tumor cell death through two different mechanisms. First, they stop the repair of SSBs by catalytically inhibiting the PARP enzyme. Second, PARP trapping result in PARP-DNA complexes being physically locked onto the DNA strand. During the S-phase of the cell cycle, these trapped complexes function as barriers that cause collapse of replication forks, and conversion of unrepaired SSBs into deadly cytotoxic DSBs (7).

Double-strand breaks (DSBs) are repaired mainly by Homologous recombination repair (HRR) pathway during S/G2 phase of the cell cycle with the homologous sister chromatid as a repair template for DNA synthesis (8). BRCA1 and BRCA2 and other HRR related genes such as ATM, PALB2, RAD51, and CHEK2 are the key genes involved in this process (9). Mutations in these genes impair this repair mechanism, leading to the accumulation of genetic errors and an increased risk of cancer. Homologous recombination deficiency (HRD) is characterized by the inability of a cell to use HRR pathway to repair DNA DSBs (3). HRD can cause reliance on error prone pathways and can be repaired by Non-Homologous End Joining (NHEJ), in which non-Homologous Ends are joined. However, NHEJ repair can lead to deletions or insertions because the DNA ends are modified before joining (8).

**Figure 1:**
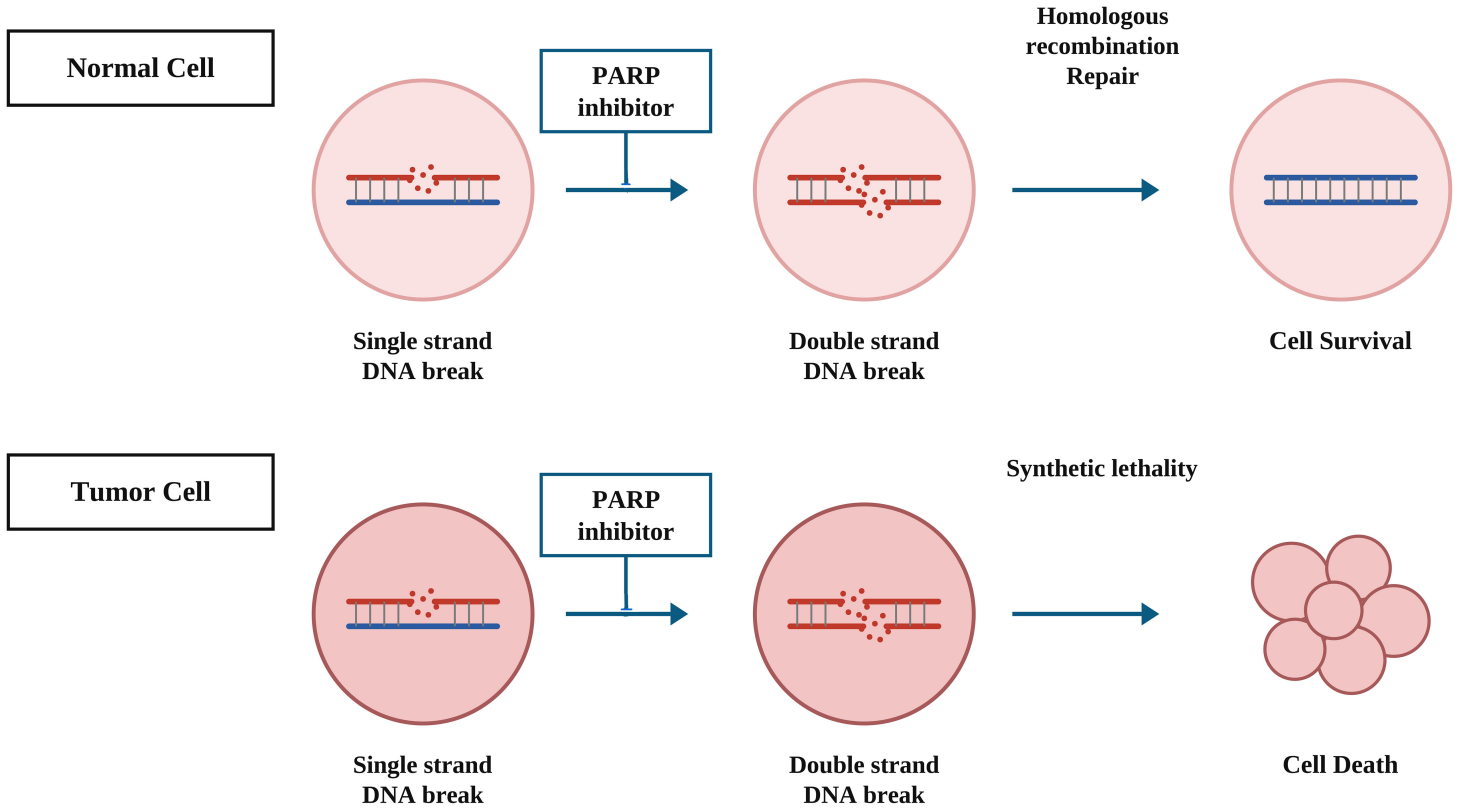
PARP inhibition in normal and HRR-deficient mutant cells. In normal cells, DNA damage could be repaired due to homologous recombination, while in HRR-deficient cells, it cannot be repaired and accumulate DNA damage, resulting in synthetic lethality. Created in https://BioRender.com.

The concept of synthetic lethality was first described in Drosophila model organism in the 20th century (10). Synthetic lethality, in which the disruption of the two complementary DNA repair pathways leads to cell death, whereas the loss of a single gene defect might have no or little effect on cell survival. The loss of functional HRR in HRD tumors causes dependency on SSB repair via PARP1. Pharmacological inhibition of PARP can selectively impair cancer cells by blocking PARP1 activity and trap it at DNA damaged sites. Unrepaired SSBs encounter DNA replication forks converting into DSBs. As a result, apoptosis is triggered through stages of chromosomal rearrangements, micronucleus production, and catastrophic genomic instability This effective DNA repair mechanism offers a therapeutic window by targeting cancer cells only while preserving healthy tissues (11). The impairment of this HRR pathway creates a therapeutic vulnerability (**Figure 1**). In HRD tumors, inhibition of poly(adenosine diphosphate [ADP]–ribose) polymerase (PARP) pathway provides a targeted therapeutic strategy (12, 13).

Poly (ADP-ribose) polymerase inhibitors (PARPi) are a class of drugs that target both PARP1 and PARP2 referred to as PARP1/2 inhibitors that has been effective in HR deficient tumors. They have been approved for treatment of several solid tumors such as BRCA-mutated ovarian, pancreatic and breast cancer. The first-generation PARPi including Olaparib, rucaparib, niraparib, and talazoparib revolutionized the treatment for solid tumors (**Table 1**). Olaparib, was first approved in 2014 (14), followed by rucaparib in 2016 (15), niraparib in 2017 (16), and talazoparib in 2018 (17). Veliparib has not been approved, with clinical trials still going on (18). Out of these, talazoparib is considered the most clinically potent with the lowest approved dosage (17). Clinically these agents are effective with widespread success to target a variety of cancers, such as breast cancer, pancreatic cancer, prostate cancer, and ovarian cancer (17). The currently approved first-generation dual PARP1/2 inhibitors and their approved clinical indications are summarized in **Table 1**.

**Table 1:** Approved first-generation dual PARP 1/2 inhibitors in BRCA-mutated and HRR-deficient solid tumors.

Drug	PARP class	Approval year	Tumor type	Line of therapy	Reference
Olaparib	PARP1/2 inhibitor	2014	Ovarian Cancer	Platinum-sensitive, relapsed high-grade serous ovarian cancer in patients with a BRCA1 or BRCA2 (BRCA1/2) mutation	(26)
Rucaparib	PARP1/2 inhibitor	2016	Metastatic castration-resistant prostate cancer (mCRPC)	Pretreated (post-androgen therapy + chemo)	(15, 27)
Niraparib	PARP1/2 inhibitor	2017	Ovarian cancer	Maintenance treatment for patients with platinum-sensitive, recurrent ovarian cancer	(28, 29)
Talazoparib	PARP1/2 inhibitor	2018	Metastatic castration-resistant prostate cancer (mCRPC)	Pretreated, one or two taxane-based chemotherapy	(17, 30)
Veliparib	PARP1/2 inhibitor	Ongoing clinical trials	Ovarian cancer	Pretreated ≥2 previous platinum-based chemotherapy	(18, 31)

BRCA-mutated cancer patients are mostly benefitted from this, but recent clinical trials have found that non-BRCA-mutated cancer patients can also benefit from these agents. The therapeutic efficacy of PARPi bridges the gap between the BRCA mutations and Homologous Recombination Repair (HRR) deficiency. In trials such as OPINION (NCT03402841) and L-MOCA (NCT03534453) have shown that olaparib provides significant benefit in patients with platinum sensitive ovarian cancer regardless of BRCA status (19, 20).

Similarly, OReO study (NCT03106987) and PRIME trial (NCT03709316) highlight the potential therapeutic efficacy of olaparib and niraparib in different molecular profiles and ethnicities. Furthermore, a latest phase II study (NCT03344965.) has shown that PARP inhibition is effective not only in ovarian but in metastatic breast cancer as well, targeting both BRCA and HRR deficiency (21). Furthermore, a study published in Lancet Oncology July 2022 (NCT03404960), demonstrated that combining immunotherapy nivolumab with PARPi niraparib have shown effectiveness among pancreatic cancer patients, although BRCA mutation rate in pancreatic cancer is about 4-7% (22). Collectively, these data highlights that PARPi not only targets germline carriers, requiring better biomarker strategies to identify all patients with HRR-related vulnerabilities who may benefit from treatment.

Despite their remarkable clinical impact PARP1/2 inhibitors are associated with clinically significant hematological toxicities. These agents target both PARP1 and PARP2 and evidence suggests that although PARP1 inhibition is required for anticancer effect. PARP2 inhibition is considered as a major contributor to hematological toxicities such as anemia, thrombocytopenia, and neutropenia, however, talozoparib, the dual PARPi, that has shown high potency to trap PARP1, also contributed to myelosuppression. This suggests the mechanism to be related to multifactor rather than just PARP2 mediated (23). Due to these toxicities, dose reduction, interruptions, and discontinuation of treatment is required which can compromise clinical efficacy (24, 25).

Despite clinical benefits and improved tolerability as compared to standard chemotherapy, PARP 1/2 inhibitors have undesirable side effects. To mitigate the dose reduction limitations, several Next-generation PARP1-selective inhibitors have emerged. These compounds designed with higher PARP1 selectivity are currently in different stages, ranging from preclinical to early phase clinical trials. The first discovery of a potent and selective PARPi, 2-[1-(4,4-Difluorocyclohexyl)piperidin-4-yl]−6-fluoro-3-oxo-2,3-dihydro-1H-isoindole-4-carboxamide (NMS-P118), exhibited a pharmacokinetic profile that demonstrated high efficacy in breast cancer and pancreatic cancer xenograft models in preclinical studies, however, has not yet been advanced to clinical phase (32). In BRCA-mutated tumor models, AZD5305 (saruparib) demonstrated 500-fold selectivity for PARP1 over PARP2 with minimal effects on hematologic factors. AZD5305 has the ability to specifically target cancer cells with HRR deficiency while sparring cells that are HRR proficient could potentially lead to improved therapeutic index (24).

## 2 Structural determinants of PARP1 selectivity

### 2.1 Catalytic domain architecture of PARP1 *vs* PARP2

PARP1 and PARP2 share a common enzymatic function due to the presence of a preserved C-terminal catalytic (CAT) domain, which plays a role in the synthesis of poly-ADP-ribose (PAR) chains using nicotinamide adenine dinucleotide (NAD+) as a donor (33). This shared catalytic activity is mediated by conserved structural features within the catalytic domain. The ADP-ribosyl transferase (ART) domain is the structural region that contains the NAD+ binding site responsible for the shared catalytic activity in PARP enzymes (34). The ART domain is composed of β-α-loop-β-α signature motif and the H-Y-E catalytic triad that is critical for the initiation, elongation, and branching of PAR synthesis (35).

A helical domain (HD) regulates the function of the ART domain by preventing NAD+ from binding to the active site, controlling the catalytic activity through conformational change. Once DNA binds, helices αB and αF within the HD become unstable allowing the entry of NAD+ and a kink is formed in helix αF (D766 in PARP1/E335 in PARP2) (36).

Although the structure of PARP enzymes is conserved, they differ in their N-terminal DNA binding modules. PARP2 has only two nucleic acid binding domains (disordered with highly charged N-terminal region and WGR domain. PARP1 has three zinc fingers and a BRCT domain that allows the binding of different nucleic acid structures such as single-stranded breaks, double stranded breaks, and G-quadruplexes. These enzymes require direct contact between the WGR domain, and 5’-phosphorylated DNA ends for allosteric enzyme activation (37).

PARP1 and PARP2 have high sequence similarity (90% similarity and 84% identity) in their catalytic domains, that leads to a major challenge which is increasing the selectivity of inhibitors in drug development (38).

### 2.2 Structural basis of selectivity: the TY-pocket

The main difference between PARP1 and PARP2 structure is the presence of a hydrophobic region adjacent to the nicotinamide binding site in PARP1 called TY-pocket. This is due to a residue of geometry substitution. There are three key common interactions by the first-generation inhibitors which are hydrogen bonds, and π-stacking interactions such as Tyr907 in PARP1 (39). Tyr907 is critical residue in the interaction and binding of PARPi, however, its phosphorylation can lead to drug resistance in Veliparib, Olaparib, and Rucaparib, by reducing the binding affinity of inhibitors and increase PARP1 catalytic activity.

An example of a PARP1-selective inhibitor is AZD5305, which binds to NAD^+^ binding pocket through quinazolinone scaffold forming hydrogen bonds that stabilize the backbone atoms. The interaction of amino acids with PARPi facilitates the stronger and selective binding to the active site (40). In PARP2, cysteine (Cys) is in an equivalent position to Tyr907, which provides an electrostatic environment interfering with the binding of next-generation inhibitors. Studies have shown that the 4′4-difluorocyclohexyl ring on PARP1 inhibitor called NMS-P118 can form hydrophobic interactions with Y889 resulting in a stronger binding of PARP1 (41). In addition, H862 is known to have a stronger free energy binding in PARP1 than H428 in PARP2. This difference is due to the presence of stronger hydrogen bonds and shorter distances.

**Figure 2:**
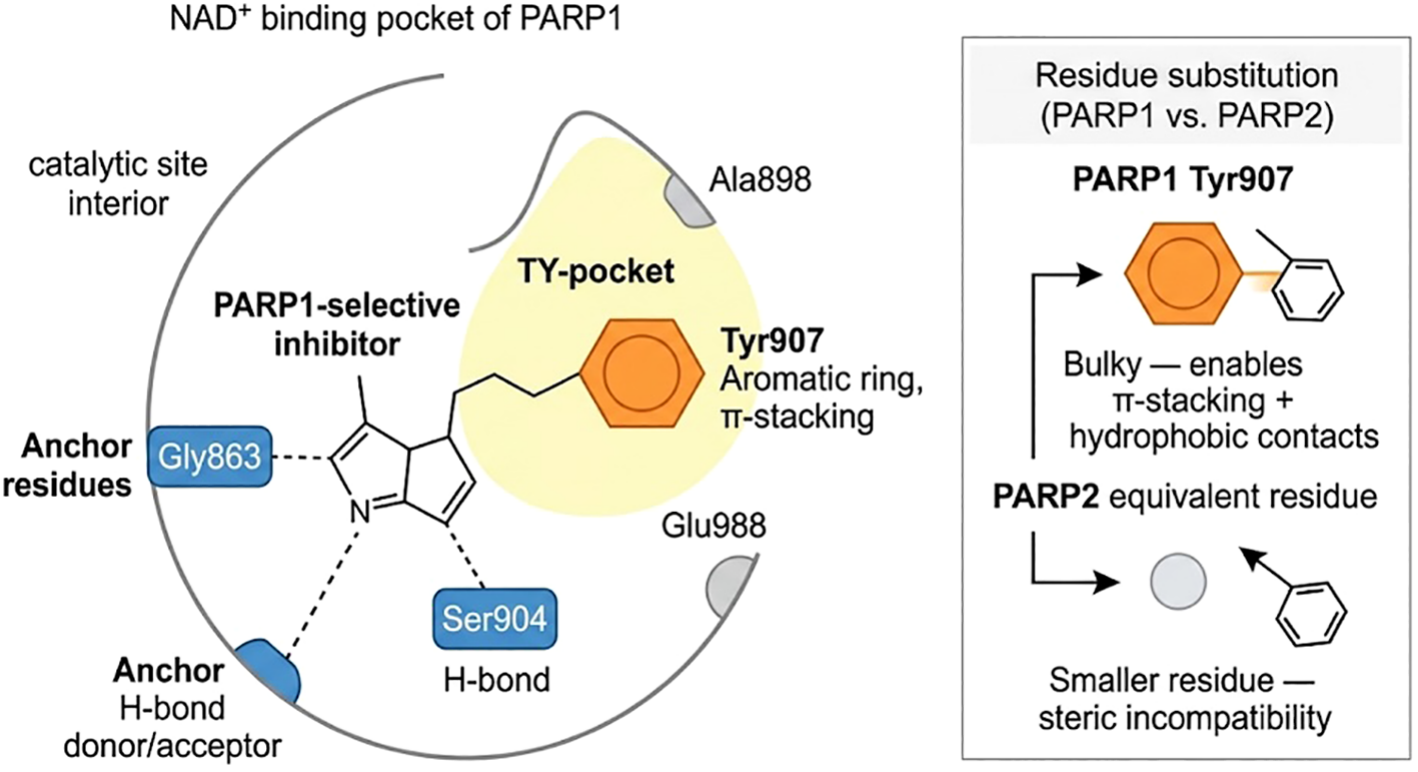
Representation of PARP1 NAD+ binding pocket and selectivity determinants.

To represent the interaction of the structural basis of PARP isoform selectivity and the hydrophobic TY-Pocket, (**Figure 2**) demonstrates the differences of PARP1 from PARP2. The TY-Pocket is defined by the bulky Tyr907 residue that enables π-stacking and hydrophobic interactions with selective inhibitors. However, the same position is occupied in PARP2 with Cys residues. Targeting these unique differences can allow next-generation inhibitors to have high specificity and avoid resistance.

### 2.3 PARP trapping and allosteric effects

#### 2.3.1 Mechanism of PARP trapping

There are specific assays to monitor PARP1 retention on damaged DNA through two step mechanisms. The first primary step is through catalytic inhibition by competing with NAD+ for the same binding site. This is followed by allosteric modulation of the bound inhibitor, where each PARPi behaves distinctly either by trapping PARP1 on DNA or promoting its release (42). This mechanism of PARP trapping leads to the formation of cytotoxic PARP-DNA complex that will interfere with DNA replication process causing stress and inducing cancer cell death (43).

#### 2.3.2 Reverse allostery and differential trapping

PARPi are categorized into three types based on each allosteric effect. Type I inhibitors promote longer attachment to the DNA, called pro-retention allosteric effect. It works by increasing the dynamics of HD helices, changing the protein shape to have better interaction with WGR domain which is critical for DNA binding and recognition. These reverse allosteric effects differ between PARP1 and PARP2. For example, talazoparib, olaparib, and niraparib act as Type I inhibitors in PARP2, however, veliparib has the same effect in both enzymes, where it acts as a Type III inhibitor (44).

The compound AZD5305 is designed for PARP1-selectively and still can cause pro-retention to reverse allosteric effect on PARP2. In the HD both enzymes have differences in helix F positions, which explains the allosteric effect variations of PARPi (44).

#### 2.3.3 Selective *vs* dual inhibitors: trapping potency

PARPi reverse allosteric effect on PARP1 DNA binding and enzymatic inhibition, which restricts PAR synthesis and prevent self PARylation (the process of using NAD+ to synthesize PAR chains) that acts as release signal from DNA. Although PARP1 affinity is one order of magnitude lower, Veliparib at 100 μM cannot replicate the trapping effect seen in 1 μM of talazoparib, indicating that allosteric properties can control the trapping formation rather than the catalytic potency (45).

The compound AZD5305 exhibits less hematologic toxicity in rats when compared to dual PARP1 and PARP2 inhibitors such as Olaparib and niraparib. They show strong PARP1 DNA trapping activity with IC50 having value of 3 nM for PARP1 and 1400 nM for PARP2, with no obvious effect hematologic and bone marrow cells (24).

### 2.4 Implications for rational drug design

First-generation inhibitors were designed to be selective; however, the PARP proteins are structurally very similar and additional interactions are needed to selective inhibition. Clinically, PARPi are very active, and small concentrations are enough to inhibit PARP1 and PARP2 together. This selectivity of these enzymes over other PARP enzymes is due to the formation of water mediated hydrogen bonds with D766 residue present in PARP1/2 but not the others (46).

First-generation PARPi were not designed for high selectivity of PARP1. AZD5305 is PARP1-selective and developed as trapper with low hematologic toxicity in comparison to first-generation inhibitors (24, 43).

Current clinically approved PARPi are classified as first-generation and have non-selective binding and inhibitory effect of PARP1/2. The selective inhibition of PARP1 only is sufficient to cause cell death in homologous recombination deficient cancers, however, PARP2 is associated with hematological toxicity (47).

The compound AZD5305 was developed based on structure and properties that includes screening of PARPi (selective for PARP1/2), x-ray crystallography, parallel chemistry to create analogs, and nicotinamide-mimetic core (48). AZ4554 is the lead compound that acts as PARP1-selective and DNA trapper in BRCA-mutant cells. To create AZD5305, polar atoms were introduced to lower logD without reducing its oral bioavailability or permeability (24, 48).

Studies have used molecular dynamics simulations to compare the interaction of selective (NMS-P118 and saruparib) and non-selective (olaparib and veliparib) PARPi with PARP1 and PARP2. They found that selective inhibitors can interact with PARP1 in a specific atomic pattern, saruparib shows up to 500-fold selectivity that was translated to clinical trials revealing real therapeutic benefits in HR-deficient tumors (49). The similarity of PARP1 and PARP2 in structure is a central challenge to achieve molecular selectivity for PARP1 inhibitors. If the inhibitor lacks selectivity to distinguish between PARP enzymes, both will be inhibited increasing hematological toxicity and limiting the therapeutic efficacy. NMS-P118 showed around 150-fold selectivity over PARP2 and remained preclinical, while saruparib revealed more favorable properties of selectivity and safety that allowed its advancement to clinical applications (49).

PARP2 inhibition is associated with hematological toxicity that can affect the monotherapy and combination therapies. In BRCA-mutated cancer cells, PARP1 inhibition creates synthetic lethality, whereas PARP2 inhibition is not essential in cancer cell death. Providing the biological basis for the development of next-generation inhibitors (PARP1-selective) (50). The major structural and functional differences between PARP1 and PARP2 are summarized in **Table 2**.

**Table 2:** PARP1 vs PARP2: structural and functional comparison.

Feature	PARP1	PARP2	Key reference(s)
Molecular weight	113 kDa	62 kDa	(36)
N-terminal DNA-binding domains	ZnF1, ZnF2, ZnF3, BRCT, WGR	Disordered NTR, WGR	(36)
DNA substrates recognized	SSBs, DSBs, G-quadruplexes	5′-phosphorylated SSBs	(33)
Catalytic domain sequence identity	—	84% identical/90% similar to PARP1	(51)
HD regulatory kink residue	D766 (helix αF)	E335 (helix αF)	(36)
Selectivity-determining residue	Tyr907	Cys (equivalent position)	(49)
Contribution to cellular PAR synthesis	85–90%	10–15%	(35)
Role in synthetic lethality (BRCAm)	Primary	Dispensable	(50)
Associated toxicity upon inhibition	Antitumor effect	Hematological toxicity	(47)

## 3 PARP2 inhibition as a driver of hematologic toxicity

### 3.1 Role of PARP2 in erythropoiesis

Erythropoiesis is a complex process in which hematopoietic stem cell gives rise to mature red blood cells (RBCs). This process must be tightly regulated to maintain RBCs synthesis under both normal and stress conditions controlled by multiple proteins. Impairment of this pathway can cause anemia and can be fatal under stress conditions (52).

In BRCA-mutated cancers, synthetic lethality is caused by PARP1 inhibition and trapping while PARP2 may not be involved in anticancer activity (53). In mice models, PARP2 has been demonstrated to play a significant role in the survival of hematopoietic cells which suggests that inhibiting and trapping PARP2 may lead to hematologic toxicity as observed clinically (54).

PARP2 plays a significant role hematopoietic stem/progenitor cells (HSPC) survival. A study by Farrés et al. has shown that chronic anemia had been developed in PARP2 deficient mice (Parp2−/−) along with reduced erythroid progenitor cells in bone marrow (54). In the absence of PARP2, unresolved DNA damage accumulated during S phase, causing cell cycle arrest and apoptosis leading to depleted erythroid progenitor pool and impairing RBCs output (55).

The transcription factors involved in the regulation of erythroid cells include GATA-1, GATA-2, NF-E2, SCL/tal-1, AML1, Myb, PU1 and rbtn2 (56). GATA1 belongs to the GATA family. It is considered the major transcription factor in erythropoiesis by regulating erythroid maturation and function at transcription level. The GATA1 protein consists of two zinc finger domains and an N-terminal transactivation domain. GATA1 protein is highly regulated process at different levels in erythroid cells such as transcription, mRNA translation, posttranslational modifications, and protein degradation. GATA1 translation produces the protein with a shorter variant lacking transactivation domain that is functionally deficient in erythropoiesis. High GATA1 protein levels are required for early stages of erythroid maturation while downregulating GATA1 protein level is essential in terminal erythroid differentiation. Maintaining GATA1 protein levels is significant for its homeostasis with its overexpression leading to lethal anemia (57).

Along with erythropoiesis, PARP2 inhibition has also been associated with impaired proliferation and maturation of megakaryocyte (43). In ovarian cancer studies, thrombocytopenia has been observed to rise during the first cycle, and then plateaus after the second and third cycle suggesting reversible inhibition rather than irreversible bone marrow suppression (58).

### 3.2 Impact on hematopoietic stem and progenitor cells

PARP2 has a significant role in hematopoietic stem/progenitor cells (HSPCs). HSPCs maintain the hematopoietic system within the bone marrow and gives rise to all mature red blood cells. They are highly dependent on cell cycle checkpoints, apoptosis, and DNA repair to maintain hematopoietic homeostasis (54). Previous studies have shown that they are more resistant to ionizing radiation, as compared to other subsets of hematopoietic cells. Ionizing radiation can lead to NHEJ pathway for DNA repair in HSPCs for survival whereas leads to apoptosis in their downstream progeny (59). DNA repair and genomic stability is required to maintain hematopoietic stem cell function, as it can be caused due to the accumulation of DNA damage in HSPCs and impairment of these pathways could lead to hematopoietic malignancies and bone marrow failure (60). Thus, exposure to toxic agents such as ionizing radiation above threshold leads to the loss of HSPCs causing hematological toxicity side effects in treated patients. Hence understanding the DNA damage response in HSPCs is significant for improved clinical management of treatment associated side effects.

To evaluate the side effects and toxicity, a study by Hopkins et al. used ex vivo colony formation assay using hematopoietic progenitors derived from human bone marrow. They compared the cytotoxic potency of multiple first-generation dual PARP1/2 inhibitors such as olaparib, veliparib, rucaparib, and talazoparib at concentrations respective their therapeutic ranges towards erythroid and myeloid progenitors. The comparison between IC50s for PARP catalytic inhibition in bone marrow mononuclear cells (BMN) showed 40-fold difference between the most potent and least potent PARPi while the cytotoxicity of BMN were 600 folds. For veliparib, olaparib, and rucaparib showed greater than 100-fold ratio between PARP inhibition and bone marrow toxic concentrations, whereas for the potent talazoparib the IC50 difference between PARP inhibition and bone marrow cytotoxicity was less than 2 folds. Interestingly, talazoparib was 73 times more cytotoxic against hematopoietic progenitor cells than rucaparib (23). The narrow therapeutic index of Talazoparib aligns with the meta-analysis data reported by Maiorano et al., with key clinical observations of talazoparib having the highest rate of hematologic toxicity among all approved PARPi. Across all 31 randomized trials, Talazoparib reported the highest relative risk for all grade and  ≥ G3 Anemia (RR 3.33 and 10.23 respectively) (58). These findings suggest that PARP inhibition is a key driver in bone marrow toxicity associated with PARPi treatment associated side effects.

NMS-P118 is a first potent and selective PARP1 inhibitor that has shown anticancer activity both in monotherapy and in combination (32). Another study by Illuzi et al. demonstrated that PARP1 alone would be sufficient for anticancer activity. AZD5305, next-generation PARPi is a highly potent and selective PARP1 inhibitor, with 500-fold selectivity for PARP1 over PARP2. In rat pre-clinical models, it achieved elevated tumor suppression at doses ≥0.1 mg/kg once daily as compared to olaparib with much higher dose of 100 mg/kg once daily. It also caused minimal hematological toxicity at predicted clinical doses as compared to first-generation dual PARP1/2 inhibitors (24). These findings support the hypothesis that targeting PARP1 alone may be sufficient for anticancer activity while reducing hematological toxicity caused by PARP2 but confirmation from clinical studies is still required.

Similarly studies by Ronson et al. and Murai et al. have shown PARP1 loss as key driver in synthetic lethality in HRD with trapping PARP2 not necessarily required for synthetic lethality (53, 61). *In vitro* hematological toxicity assay demonstrated AZD5305 had less toxicity on HSPCs than talazoparib (62). These findings collectively provide the mechanistic framework for PARP2 significant role in proliferation and differentiation of HSPCs while not necessarily required for anticancer synthetic lethality, highlighting it as an unnecessary toxicity target of the current first-generation dual 1/2 PARPi. The reported incidence of grade ≥3 hematologic toxicities associated with currently available dual PARP inhibitors is summarized in **Table 3**.

**Table 3:** Hematological toxicities grade ≥ 3 of standard dual PARPi.

Drug	Trial	Therapy line	Anemia ≥ G3	Thrombocytopenia ≥ G3	Neutropenia ≥ G3	Reference
Olaparib	SOLO1	First line, ovarian cancer	22%	<1%	8%	Banerjee et al., 2021 (63).
Olaparib	SOLO2	platinum-sensitive, recurrent ovarian cancer.	19%	1%	4%	Pujade-Lauraine et al., 2017 (26).
Rucaparib	ARIEL3	platinum-sensitive, recurrent ovarian cancer.	18%	3%	5%	Coleman et al., 2017 (64).
Niraparib	NOVA	platinum-sensitive, recurrent ovarian cancer.	25.3%	33.8%	19.6%	Mirza et al., 2016 (28).
Talazoparib	EMBRACA	advanced breast cancer, ≥2 prior lines of platinum-based chemotherapy	55%	11.2%	17.8%	Litton et al., 2018 (65).
Veliparib	NRG Oncology/GOG study	primary ovarian, fallopian tube, or primary peritoneal cancer, ≥2 prior lines of platinum-based chemotherapy	<1%	<1%	1%	Coleman et al., 2019 (31)

G3, grade 3 or higher, Veliparib is not FDA-approved. Rate of toxicity reported for the treatment arm only.

### 3.3 Preclinical evidence supporting selective targeting

Currently all approved PARPi have non-selective binding and inhibition of both PARP1 and PARP2. However, inhibition of PARP1 has been shown to be sufficient for the HRR-deficient cancer cells with PARP2 inhibition being redundant for cancer cells. PARP2 trapping and inhibition has been associated with hematological toxicity that has been observed in treatment associated side effects of PARPi. A study by Staniszewska et al. tested AZD9574, a blood brain barrier penetrant selective PARP1 inhibitor alongside olaparib on HSPCs. They used hematological toxicity assay and demonstrated that AZD9574 at dose achieving inhibition of PARP caused less hematological toxicity as compared to olaparib. Olaparib caused reduction in viability with a decrease of up to 25% at 167 to 330 nmol/L and an IC50 of 0.75 mmol/L while AZD9574 showed 25% decrease maintained across a wide range of concentration with IC50 of 4.13 mmol/L (66).

Similarly, *in vivo* rat models, as a monotherapy AZD9574 was well tolerated without any hematological toxicity across the tested dose range. However, in combination therapy, AZD9574 was used with temozolomide, a brain-penetrant alkylating chemotherapy. The hematological toxicity on the erythroid, platelets, and neutrophil was observed in a dose dependent manner. Treatment with 10 mg/kg TMZ had effects on neutrophils and platelets and was not well tolerated. The effects were mitigated by lowering the dose of both TMZ to 5 mg/kg and AZD9574 from 3 to 1 mg/kg (66).

In another study, rat models were treated for 14 days with selective PARP1 inhibitor AZD5305–1 mg/kg once daily and first-generation dual PARP1/2 inhibitor olaparib 100 mg/kg once daily or niraparib at 57 mg/kg once daily. As reported clinically, consistent reduction in in immature red blood cells were observed, in contrast AZD5305 had reduced hematological toxicity. Similarly, treatment with niraparib also resulted in reduced reticulocytes and slight reduction in neutrophil count. These results highlight selectively targeting PARP1 mitigate the hematological toxicity that is observed in dual PARP1/2 inhibition (24).

Along with monotherapy, AZD5305 (1 mg/kg) was also investigated in combination with one cycle of intravenous carboplatin (30 mg/kg) in rat models. As a result, stable hemoglobin levels and red blood cell counts were observed. In histopathologic analysis of the bone marrow, with exception of 1 out of 8 animals, which had minimal decreased cellularity of bone marrow was observed. In contrast, olaparib + carboplatin treatment showed reduction in hemoglobin levels and red blood cell that did not recover, with histopathology analysis further confirming increased incidence and severity of decreased bone marrow cellularity. Next a two-cycle study that used a higher carboplatin dose replicated the findings, with AZD5305 demonstrating recovery of reticulocyte counts between treatment cycles. These findings could allow chemotherapy at full therapeutic dose in combination with selective PARPi (24). However, one of the limitations is that the evidence present relies heavily on preclinical *in vitro* and *in vivo* models. Although this evidence has demonstrated the basis of contribution of PARP1 and PARP2 in hematological toxicity but may fully not reciprocate the complexity of human bone marrow. Thus, clinical confirmation in large cohorts is essential to consider these preclinical findings definitive.

Beyond these limitations, several unresolved questions remain regarding highly selective PARP1 inhibition. First PARP1 and PARP2 together has redundant role in Base excision repair (BER) (53). So selectively sparing PARP2 may reduce accumulated DNA repair burden caused by dual inhibition on tumor cells. This raises a possibility that in exchange of improved tolerability, some of the antitumor efficacy contributed by PARP2 could be sacrificed. Second, PARP1-selective inhibitors such as AZD5305 long term efficacy data are limited to early phase trials (67). Thus, the response durability over prolonged treatment period is not yet clear. Finally, as PARP1-selective inhibitors become more widely used in clinical settings, it is required to characterize and overcome real world resistance patterns in larger and more diverse patient populations.

## 4 Clinical translation of PARP1-selective inhibitors

### 4.1 Saruparib (AZD5305) as a prototype agent

First-generation PARPi have demonstrated significant success in cancer therapy; however, they exhibit limited selectivity, as they inhibit both PARP1 and PARP2. This lack of specificity has led to considerable clinical challenges, since inhibition of PARP2 has been associated with increased toxicities—most notably hematological toxicities such as anemia, neutropenia, and thrombocytopenia (62). In one study, knockout of PARP2 in mice resulted in increased cell death in hematopoietic stem/progenitor cells (HSPCs) and bone marrow failure (54), further demonstrating the link between PARP2 inhibition and heightened hematological toxicity. Therefore, selective inhibition of PARP1 is crucial for minimizing these adverse effects. Moreover, PARP1 has been shown to play a more dominant role in cellular PARylation compared to PARP2, accounting for approximately 80–95% of total PARylation activity, with PARP2 contributing the remaining 5–20% (24). This prompted researchers to come up with a highly selective PARP1 inhibitor saruparib (AZD5305).

AZD5305 work by inhibiting the catalytic activity of the PARP1 enzyme through a trapping mechanism, in which the PARP enzyme remains bound to damaged DNA. This trapping causes double-strand breaks, ultimately leading to cell death (24, 61). According to one study, AZD5305 exhibits higher selectivity for PARP1 over other PARP family members, reaching almost 500-fold selectivity (62). Another research paper evaluated the antitumor activity of AZD5305 compared to olaparib and found that AZD5305 demonstrated superior efficacy, however clinical validation remains limited to early phase studies. Specifically, AZD5305 achieved a 75% complete response rate, compared to 37% for olaparib. In terms of progression-free survival, AZD5305 also produced more favorable outcomes 386 days vs. 90 days for olaparib (67). Moreover, AZD5305 displays improved hematological tolerability relative to first-generation dual PARP1/2 inhibitors. It was shown to cause less hematological toxicity than talazoparib—a first-generation inhibitor—when assessed in human *in vitro* hematopoietic stem/progenitor cells (HSPCs) (62). Similarly, a study by Herencia-Ropero et al., has further independently confirmed the antitumor activity of AZD5305 (saruparib) compared to first-generation olaparib in patient derived BRCA 1/2 xenograft models. It was further demonstrated that the antitumor activity with acquired resistance could be overcome by combination treatment such as combining with carboplatin or the ATR inhibitor ceralasrtib. These findings suggest the efficacy of saruparib and adds confidence of its reproducibility across different laboratory settings (67).

### 4.2 Clinical activity across HRR-deficient tumors

Furthermore, PARPi are known to be most effective in patients with ovarian, breast, pancreatic, or prostate cancers that harbor mutations in homologous recombination repair (HRR) pathway genes, including *BRCA1*, *BRCA2*, *PALB2*, and *RAD51* (68). Illuzzi et al. examined the antiproliferative effects of AZD5305 alongside first-generation inhibitors such as olaparib, niraparib, and talazoparib, in paired cell lines carrying either mutant or wild-type versions of the *BRCA1*, *BRCA2*, *PALB2*, and *RAD51C* genes (24). It was shown that AZD5305 was markedly more potent in inhibiting proliferation in mutant cell lines compared to their corresponding wild-type pairs, which displayed minimal effects. There was over a 5 log (100,000 times) difference in efficiency between the two cell types. Although similar trends were observed with first-generation PARPi, the difference between wild-type and mutant cell lines was smaller; around two to three-log (24). These results highlight the high selectivity of AZD5305 toward HRR-deficient (HRD) cell lines compared to HRR-proficient wild-type cells, demonstrating superior antiproliferative activity relative to first-generation inhibitors.

### 4.3 Emerging next-generation inhibitors

While AZD5305 (saruparib) continues to be the most studied due to its advanced clinical state, several next-generation agents are emerging with similar preclinical characteristics. AZD9574, a selective PARP1 inhibitor has ability to penetrate blood brain barrier. In HSPCs based assay, it has also demonstrated reduced hematological toxicity as compared to olaparib. It has shown favorable tolerability in both monotherapy and in combination with the CNS penetrant chemotherapeutic agent temozolomide in rat models (66). Similarly, multiple next-generation PARP1 inhibitors have emerged, including KBD111 (69), CKD-9001 (70), and HSK46256 (71). These inhibitors exhibit higher selectivity toward PARP1 and demonstrate strong potency, along with the ability to penetrate the central nervous system (CNS). Their high blood–brain barrier (BBB) permeability suggests potential utility in the treatment of brain tumors and CNS metastasis (69–71). All three compounds exhibited promising preliminary pharmacokinetics properties, decreased toxicity toward hematopoietic stem cells, and stronger tumor growth inhibition in *BRCA*-mutant or HRD models compared to other PARPi such as olaparib (69–71). However, unlike AZD5305 (saruparib), these agents remain in preclinical trials, and their safety, efficacy as well as pharmacokinetic profiles require further evaluation in human clinical trials before comparative clinical benefits can be concluded.

### 4.4 Redefining clinical benefit

Collectively, these preliminary findings highlight the promising therapeutic potential of next-generation PARP1-selective inhibitors, particularly in HRD models. These compounds are characterized by higher potency and selectivity toward the PARP1 enzyme, improving tolerability through reduced hematotoxicity, and enhanced antiproliferative activity compared with first-generation inhibitors. Despite these promising results, most of these findings come largely from preclinical or early phase trials. Thus, the improved therapeutic index observed in preclinical models are needed to be further evaluated to ensure its long-term clinical outcomes, safety, efficacy and improved overall survival in broader patient population. The key characteristics, clinical development status, and major findings of currently available next-generation PARP1-selective inhibitors are summarized in **Table 4**.

**Table 4:** Summary of next-generation PARP1-selective inhibitors.

PARPi	PARP1 selectivity	Phase	CNS penetration	Key findings	Reference
Saruparib (AZD5305)	~500-fold selective for PARP1 over PARP2	Clinical (Phase I/IIa, PETRA, PETRANHA trials)	Not primary focus	48.4% ORR at 60mg in HRR-deficient breast cancer; resistance overcome with carboplatin or ATR inhibitor combination	(24, 67, 72)
AZD9574	PARP1-selective	Preclinical to early clinical (glioma trials ongoing)	Yes	Reduced hematologic toxicity compared to olaparib in HSPC assays; superior survival compared to temozolomide alone in MGMT methylated glioma models	(66)
KBD111	PARP1-selective	Preclinical	Yes	Decreased HPSC toxicity; stronger tumor growth inhibition	(69)
CKD-9001	PARP1-selective	Preclinical	Yes	Similar profile to KBD111	(70)
HSK46256	PARP1-selective	Preclinical	Yes	Similar profile to KBD111/CKD-9001	(71)
NMS-P118	Potent, selective PARPi (first discovered)	Preclinical	Not reported	High efficacy in breast and pancreatic cancer xenograft models	(32)

## 5 PARP1 selectivity as an enabler of combination therapy

### 5.1 Limitations of first-generation combinations

The effectiveness of PARPi as monotherapy in homologous recombination repair (HRR)- deficient tumors demonstrates a strong rationale for exploring combination strategies, in order to enhance antitumor activity through synergistic targeting of DNA damage response pathways and to overcome primary or acquired resistance (73). From a mechanistic perspective, combining PARPi with chemotherapy or other DDR- targeted agents was anticipated to produce additive or synergistic DNA damage accumulation in HRR – deficient tumor cells that these cells could not withstand because they are already compromised in DNA repair (74). However, first-generation dual PARP 1/2 inhibitors with chemotherapy were hindered by significant overlapping hematologic toxicity in early clinical investigations, which emerged as the dose- limiting challenge across multiple combination regimens (73). In a Phase I/IB study, hematologic toxicity emerged as an adverse event observed with olaparib plus carboplatin in BRCA 1/2 mutation associated breast and ovarian cancer, with Grade 3/4 neutropenia, thrombocytopenia, and anemia occurring in 42.2%, 20.0%, and 15.6%, respectively, of patients (75). Consistent with these findings, a randomized Phase 2 trial found that olaparib plus paclitaxel/carboplatin showed higher rates of neutropenia and anemia compared with chemotherapy alone, suggesting that combined PARP inhibition amplifies platinum- induced myelosuppression (76). These hematologic adverse events necessitated dose interruptions, with neutropenia and thrombocytopenia occurring in 47% and 39% of patients, respectively, in a Phase I study of olaparib with carboplatin and paclitaxel, and causing treatment delays (77). Collectively, these results demonstrated that combining first-generation PARPi with chemotherapy at full doses cannot be implemented clinically because mandatory reduction in the doses of both agents was required for safe administration (78). Emerging evidence indicates that PARP2 inhibition is primarily responsible for this hematologic toxicity, whereas synthetic lethality is mediated by PARP1 in HRR-deficient tumors, suggesting the mechanistic rationale for next – generation PARP1-selective inhibitors as potentially more tolerable combination partners (50).

### 5.2 DDR-targeted combinations

In contrast to cytotoxic chemotherapy, which causes broad and non-selective DNA damage, DDR targeted agents induce DNA damage in cancer cells that carry DDR gene deficiencies, providing a mechanistically rational and potentially less myelosuppressive combinatorial approach with PARPi (79). Among DDR targeted agents, a combination of ATR(ataxia telangiectasia and Rad-3 related kinase) inhibition with PARP inhibition synergistically increases replication fork stalling, DNA double–strand break accumulation, and apoptosis in HRR- deficient tumor cells (80). Importantly, the selective PARP1 inhibitor saruparib (AZD5305) in combination with ATR inhibitors has demonstrated reduced myelosuppression; in PDX models, saruparib combined with the ATR inhibitor ceralasertib produced complete tumor regression even in PARPi resistant models, with a safer tolerability profile (67). Another rational target for a DDR combination is WEE1 inhibition, which inactivates the G2/M checkpoint, and particularly in TP53-mutated tumors that have already lost the G1/S checkpoint, results in irreparable DNA damage and apoptosis when combined with PARP inhibition (81). Clinically, the phase II EFFORT trial demonstrated that adavosertib, a WEE1 inhibitor combined with olaparib, showed clinical efficacy in PARPi resistant patients, yielding a 29% of objective response rate and 6.8 months of progression-free survival, compared with 23% and 5.5 months, respectively, with adavosertib monotherapy, in PARPi resistant ovarian cancer patients (81). Nevertheless, hematologic toxicity associated with PARP2 inhibition limits combination tolerability for DDR combination regimens, and next-generation PARP1-selective inhibitors, while maintaining synthetic lethality, represent a more promising backbone for ATR and WEE1 inhibitor based combination strategies (50).

Furthermore, combining PARPi with Receptor Tyrosine Kinase inhibitors (RTKs) represent an underexplored and promising avenue. RTK plays a significant role in regulating DNA damage and HR repair pathways. Emerging evidence has suggested that RTKs such as VEGFR, AXL, EGFR, PDGFRs, and c-MET can induce BRCAness like state. In acquired resistance to PARPi, targeting RTKs can cause synthetic lethality. The synergistic effects demonstrated are due to key molecular mechanisms such as PARP activation, DNA damage and HR repair pathway modulation, cell cycle control and apoptosis. This provides a strong mechanistic role in combining RTKs with PARP inhibition (82). Combining RTK inhibitors with the next-generation PARPi highlight the need for further preclinical and clinical investigation.

### 5.3 Chemotherapy combinations revisited

The overlapping myelosuppression that hindered the first-generation PARPi and chemotherapy combinations made them clinically unfeasible, and has generated renewed interest in revisiting their interactions with PARP1- selective inhibitors, which are expected to produce a safer toxicity profile and widen the therapeutic window to facilitate full dose combination administration (83). Preclinical evidence showed that saruparib plus carboplatin improved hematologic tolerability compared with olaparib plus carboplatin, recovering peripheral reticulocytes and myeloid erythroid precursor cells during continuous saruparib administration but not during olaparib administration (24). Compared with the first-generation dual PARP1/2 inhibitors, saruparib combined with carboplatin showed better antitumor efficacy in ovarian cancer xenograft models, stabilizing the growth of the tumor at doses as low as 0.1 mg/kg, delaying visceral metastasis, and extending the survival of tumor- bearing mice with combination treatment well tolerated (84). In consideration of these preclinical findings, a phase I/IIa modular, open-label, multicenter trial is evaluating AZD5305 as monotherapy and in combination with paclitaxel, carboplatin, and antibody-drug conjugates with the aim of characterizing its pharmacokinetic profile, tolerability, and tumor activity in patients with advanced solid tumors (43). All these preclinical and early clinical studies demonstrate that the development of PARP1-selective inhibitors represents a great advancement in the combination therapy, enabling both DDR targeted and chemotherapy based regimens that were previously limited by the myelosuppressive effects (50).

Additionally, preclinical evidence in pancreatic ductal adenocarcinoma cell lines (PDAC) has demonstrated synergistic tumor effects of PARPi olaparib, in combination with DNA damaging agents such as doxorubicin, mitoxantrone, oxaliplatin, and gemcitabine. This provides additional mechanistic role in chemotherapy based PARPi combinations (85).

### 5.4 Antibody-drug conjugates and PARP1 synergy

Antibody drug conjugates (ADCs) are powerful therapeutic agents that have gained huge attention in recent years for their ability to selectively target and treat tumors. ADCs links monoclonal antibodies with highly cytotoxic drugs through acid or enzyme labile linkers that enable controlled drug release (86). Many ADCs have demonstrated remarkable results in cancer therapy. According to the authors of one study, topoisomerase I inhibitor (TOP1i)-based ADCs show great results when combined with PARPi (87). They act synergistically to inhibit the repair of double-strand breaks, thus suppressing tumor activity. In this study, the combination of SG, an ADC with talazoparib established a median progression-free survival of 7.6 months. Sequential treatment with SG followed by a PARPi led to increased apoptosis, enhancing the tumor cell killing (87).

Similarly, researchers evaluated the combination of the PARP1-selective inhibitor AZD5305 with AZD8205, an ADC targeting *B7-H4*–expressing tumors that carries a novel TOP1i payload (88). And they found that upon adding AZD5305, the low *B7-H4*–expressing tumors became more sensitive to AZD8205 treatment, contributing to increased antitumor activity (88). Although the available data remain preliminary and require further validation, these findings suggest promising therapeutic potential for the combination of PARPi with ADCs.

### 5.5 A new combination paradigm

Research has shown that AZD5305 may provide a paradigm shift in cancer therapy, particularly when used in combination with carboplatin chemotherapy (24). The efficacy of this combination was tested across different mouse tumor models, demonstrating enhanced antitumor activity compared with monotherapy for each drug. AZD5305 monotherapy achieved 72% tumor growth inhibition (TGI), while carboplatin displayed a TGI of 88%. Notably, the combination therapy presented a significant tumor volume regression of 87% (24).

Additionally, a study by Herencia-Ropero et al. reported similar findings, showing that the combination of AZD5305 with carboplatin improved antitumor efficacy and increased tumor regression across multiple tumor models, except in those exhibiting resistance to PARP inhibition (67). The same study, also explored the combination of AZD5305 with the ATR inhibitor ceralasertib in different tumor models, which demonstrated improved tumor regression even in models unresponsive to the AZD5305-carboplatin combination without compromising tolerability (67). These results provide valuable insight into novel combination therapies that may further advance cancer treatment.

## 6 Resistance mechanisms and evolving biomarkers

### 6.1 Resistance to PARP inhibition

Despite the remarkable clinical efficacy of PARPi in HRR-deficient tumors, *de novo* and acquired resistance to PARPi represent a central obstacle to durable clinical benefit (89). Although a wide range of resistance mechanisms have been characterized preclinically and clinically, among them, a few mechanisms of PARPi resistance have been established in patients: target- level resistance, recovery of HR pathway, and stabilization of fork stability (90). Of these, the most important route of HR restoration is the reversion mutation that involves secondary somatic mutations in *BRCA 1/2*. It restores the function of HRR either through second-site deletion or insertion, or in- frame deletion of the original mutation, or through true reversion to the wild-type sequence (90). Reverse mutations in BRCA 1/2 were identified in 75 of 247,926 tumor samples across various cancer types, especially in patients with resistance to platinum based chemotherapy or PARP inhibition (91). Furthermore, the BRCA reversion mutations were prevalent in patients with prior resistance to DNA-damaging therapies, and their identification in pretreatment cfDNA (cell-free DNA) was linked to lower progression free survival upon rucaparib treatment (median 1.8 vs. 9.0 months). This demonstrates that cfDNA based detection can be established as a clinically useful tool for identifying primary PARPi resistance before treatment initiation (90, 92). Longitudinal ctDNA analysis revealed that in 60% of patients with BRCA1/2 reversion mutation who developed resistance, with multiple unique reversion mutations emerging simultaneously, alongside co-occurring mutations identified in other PARPi resistance genes (TP53BP1, RIF1, and PAXIP1) (93). The parallel emergence of multiple divergent subclones in patients indicates that acquired resistance occurs concurrently under PARPi therapeutic pressure (94). Mechanistically, BRCA reversion mutation has been largely associated with microhomology- mediated end-joining, involving POLθ enzyme encoded by the POLQ gene, which exploits end- joining of small sequence microhomologies surrounding the DNA breakpoint to restore the disrupted open reading frame (95). Beyond HR restoration, replication fork stabilization has been identified as a mechanistically distinct PARPi resistance mechanism, even in the absence of restored homologs recombination (96). In BRCA-deficient cells, stalled replication forks undergo MRE11 and EXO1-mediated fork degradation, which contributes to PARPi sensitivity (96). However, resistance emerges when GRB2-mediated stabilization of RAD51 at stalled replication forks is restored independently of HR, thereby protecting nascent DNA from MRE11-mediated degradation (97). Although these resistance mechanisms broadly operate across all PARPi, the emergence of next-generation PARP1-selective inhibitors introduces additional resistance mechanisms including PARP1 mutations, loss of PARG, and epigenetic reprogramming, that will be discussed in the following section (98). Though next-generation PARP1 inhibitors have the same mechanism; PARP1 inhibition and DNA trapping as the first-generation dual PARPi, they are expected to have the shared resistance pathways. However, the narrow profile of only targeting PARP1 has the possibility of PARP1 specific resistance mechanism.

### 6.2 PARP1-specific resistance mechanism

PARPi mediated cytotoxicity depends on PARP1 trapping, which can form cytotoxic PARP1 DNA complexes at DNA damage sites, and mutations within PARP1 that impair this trapping have been identified as an important resistance mechanism of PARPi (99). Notably, the PARP1 p.R591C mutation was found in an ovarian cancer patient with a mutation in the WGR domain, and this would be a critical residue for inter-domain communication in PARP1 (100). Structurally, the R591C mutation affects the WGR domain at the contact point with D45 and the HD domain and is predicted to impair inter-domain communication between the DNA binding and catalytic domains of PARP1, which allows DNA binding, thereby limiting PARP1 trapping efficiency (99). Beyond R591C, CRISPR screening revealed additional PARP1 mutations, including p.43delM and N329Q, all impairing PARP1 trapping function and conferring PARP1 intrinsic resistance across multiple functional domains (101). Furthermore, reduced or complete loss of expression of PARP1 represents an alternative resistance strategy, as loss of the function to bind DNA in the presence of PARPi was identified as PARPi resistance in genetically engineered mouse models (102). Although saruparib (AZD5305) demonstrated more potent and durable antitumor activity than first-generation PARPi in BRCA 1/2 and PALB2 associated PDX models, acquired resistance still emerges through higher RAD51 foci levels and HR restoration. The PARPi resistance was overcome by the addition of carboplatin or an ATR inhibitor in PDX models (67). This suggests that although selective PARP1 inhibitors do not overcome the common resistance mechanisms shared by all PARPi but their improved tolerability may allow full dose combination strategies previously restricted by hematological toxicity due to first-generation dual PARPi. This offers a practical rather than mechanistic advantage in addressing resistance. Importantly, PARP1 mutations confer distinct sensitivities to chemotherapeutic drugs compared to other PARPi resistance mechanisms such as BRCA1 reversion, 53BP1, and REV7(MAD2L2) mutation, suggesting that determining the underlying resistance mechanism in patients is crucial for guiding post-progression treatment decisions (99). Collectively, the diversity of PARP1 resistance mechanisms highlights that BRCA status alone is insufficient as a biomarker, and the urgent need for expanded biomarker strategies beyond BRCA will be examined in the following section (103).

### 6.3 Beyond BRCA: expanding biomarker strategies

Although BRCA 1/2 mutations are commonly used as a biomarker for PARPi sensitivity, not all BRCA-mutated patients exhibit PARPi-sensitive disease, and a significant percentage of non-BRCA patients harbor HRD tumors that may equally benefit from PARPi therapy (103). To capture HRD beyond BRCA mutations, a genomic instability status (GIS) or an HRD score was developed, integrating three critical biomarkers: loss of heterozygosity (LOH), telomeric allelic imbalance (TAI), and large-scale state transitions (LST), which outperforms each signature alone in distinguishing HRD from HR proficient tumors across breast, ovarian, and prostate cancers (104). The clinical validity of HRD scoring was established in major phase 3 clinical trials; PRIMA, PAOLA-1, ARIEL3, and VELIA, which demonstrated significant progression-free survival (PFS) benefits in HRD-positive patients regardless of their BRCA mutation status, thereby expanding the eligible patient population beyond BRCA mutation for PARPi therapy (105). However, an important limitation of genomic scars is that tumors that have undergone mutations restoring HR are likely to be misclassified as double-strand break repair-deficient, thereby leading to incorrect PARP sensitivity despite restored HR proficiency (106). To address this limitation, the functional RAD51 assay was established as a biomarker to measure HR functionality through the detection of RAD51 nuclear foci for predicting PARPi response, demonstrating 95% accuracy in predicting PARPi response compared to HRR gene mutation (67%) and genomic HRD analysis (71%) (107). The clinical validation of the functional RAD51 assay from the ENGOT - ov24/NSGO - AVANOVA 1 & 2 trial in 91 ovarian cancer patients demonstrated that combining the RAD51 assay with HRD testing improves specificity, while the functional RAD51 assay identifies HR proficient tumor in BRCA-mutated patients classified as HRD-positive by the companion diagnostic assay, supporting the role as an add on biomarker for HRD-classified patients (105). Beyond HRD scoring and functional RAD51 assay, a promising complementary biomarker, SLFN11 expression, has been identified as predicting PARPi sensitivity in high-grade serous ovarian cancer, capturing simultaneously cancer cell- intrinsic and immunological dispositions associated with DNA damaging agent sensitivity (108). Collectively, the transition from static BRCA centric biomarkers to dynamic functional assays and novel predictive tools highlights the critical need for marker guided patient selection techniques to optimize the therapeutic efficacy of PARP1-selective inhibitors (103).

Beyond BRCA mutations and HRD, biomarker driven patient selection may also expand the therapeutic benefit of PARP1-selective inhibitors in neuro-oncology. PARPi have been studied in glioblastoma where blood brain barrier penetration has been limited despite promising preclinical activity (109). IDH1/2 mutant gliomas represent a particularly relevant population. Mutant IDH produces oncometabolite 2-hydroxyglutarate, which suppresses homologous recombination repair, that creates BRCAness like state and cause PARP inhibition sensitivity (110). Similarly, MGMT promoter methylation status has emerged as predictive biomarker for PARPi and temozolomide in glioblastoma (109). Next-generation PARP1 inhibitors, AZD9574 has reported enhanced CNS penetration. In MGMT methylated orthotopic glioma models, AZD9574 has demonstrated superior survival benefit compared to temozolomide. They are now being clinically evaluated in IDH-mutant glioma patients in combination with temozolomide (66). These findings suggest that patient selection for PARP1-selective inhibitors in neuro-oncology could be stratified based on IDH mutation status and MGMT methylation as biomarkers, comparable to BRCA/HRD testing in solid tumors.

## 7 Future directions and clinical implications

### 7.1 Redefining patient selection

The traditional selection of PARPi therapy was depending on the status of the germline BRCA1/2 mutations; however, this might miss other HRR gene mutations in patients with HR deficiency such as PALB2, BRIP1, and RAD51C. Mutations that impair HRR increase reliance on compensatory DNA repair pathways and replication stress tolerance, which increase the sensitivity to PARPi through synthetic lethality. This suggests that the therapy selection should be guided by functional HRR deficiency rather than BRCA mutation alone. Ray et al., reports that 25-30% of patients with metastatic castration resistance prostate cancer carry HRR gene mutations; however, limitations of inconsistent timing of the test and low testing rates may lower the identification and implementation of precision therapeutic strategies (50, 111).

Recent platforms are targeting BRCA1/2 mutations and HRR gene changes by using comprehensive genomic profiling such as FoundationOne CDx. Other assays such as LOH can provide genomic scar measures to support the assessment of HRD. Molecular profiling provided PARPi recommendations for high percentage of patients in recent institutional experience, however, the exact number differ by each cohort. In addition to genomic scar assays, RAD51 nuclear foci scoring is becoming a useful indicator of HR capacity and may have higher accuracy at predicting PARP drug sensitivity more than genomic HRD scores (107, 112).

In the PETRA phase I/IIa trial, saruparib showed 48.4% ORR at 60mg daily dose in patients pretreated for HRR-deficient breast cancer, patients required dose reduction compared with 25-30% in trials approved dual PARP1/2 inhibitors (72, 113). Patient selection is affected by improved tolerability profile, patients who have been excluded from dual inhibitor trials due to poor performance, hematologic toxicity, or pretreated bone marrow, would be now treated with lower toxicity implications of PARP1-selective drugs (24).

### 7.2 Trial design innovations

Saruparib tolerability has made it easier to study combination treatments at first stages of the disease. The EvoPAR-Prostate01 trial evaluates saruparib in combination with new hormonal agent (abiraterone, enzalutamide, or darolutamide) in patients with metastatic prostate cancer. The two-cohort design of phase III randomized and placebo controlled had patients with and without HRR mutations, showing that PARP1-selective agent had greater effect over traditional biomarker limited populations (114). Another study supporting this approach is PETRANHA, indicating that saruparib is safe when combined with multiple hormonal agents showing limited hematological toxicity and reduced rates of treatment change (51).

The EvoPAR-Prostate01 study divides patients to two different groups based on their HRR mutation biomarker status which they reveal at the time of their study enrollment. The study designates around 550 patients to the HRR-mutant group while the HRR-wild-type group includes about 1,250 patients. The study design uses 1:1 randomization to create two separate groups. Each has allowed researchers to assess treatment effectiveness for both HRR-deficient patients and non-deficient patients without the need to conduct other clinical trials. The EvoPAR-Breast01 study investigates breast cancer through a randomized Phase III trial which tests the combination of saruparib with camizestrant against CDK4/6 inhibitor in addition to endocrine therapy in patients carrying BRCA1/2 or PALB2 mutations who have HR-positive HER2-negative advanced breast cancer. The study combines PARP1 inhibition with first-line treatment through a molecularly defined combination that targets both DNA repair and endocrine signaling pathways (115).

### 7.3 Expanding beyond HRR-deficient tumors

The clinical case for the development of PARPi use over BRCA1/2 is established but continuous. The PROfound trail indicated that the efficacy could be limited in specific non-BRCA HRR-mutated groups, showing that not all HRR gene mutations present the same sensitivity to PARPi. Emerging evidence shows that PARP1-selective inhibitors such as saruparib may enhance the activity in the replication associated stress, involving ATM-deficient tumors (36, 116).

Current research suggests that PARPi sensitivity can extend over canonical HR-deficient tumors. PARPi responsiveness can be enhanced through the alterations of CDK12 selectively, however, the clinical benefits seem to be heterogenous, this depends on zygosity and the type of the mutation (117). Moreover, ARID1A deficiency affects ATR that is activated in response to DNA damage checkpoint signaling. This indicates that PARPi utility might increase in patients with ARID1A tumors over patients with BRCA mutations (118).

The future directions in PARPi development include the use of anti-body drug conjugates and immune checkpoint inhibitors in combination with PARP1 selective agents, that is promoted by preclinical synergy and trials to indicate its effectiveness over BRCA mutations (119). Combination treatments using ATR, CDK inhibitors, or WEE ladder 1, are now being evaluated clinically in PARPi resistance malignancies, are expected to be formed by resistance mechanisms such as HRR reactivation and decreased PARP trapping (120). The use of PARP1-selective drugs has reduced hematologic toxicity allowing for combination treatments with chemotherapy, immunotherapy, or other targeted drugs that controls DNA replication, restoring the therapeutic of rational PARPi development (121).

## 8 Conclusion

Advancements in precision oncology with the emergence of selective next-generation PARP1 inhibitors directly addresses the limitations of therapeutic potential caused by the standard first-generation dual PARP 1/2 inhibitors. This review has highlighted the molecular mechanism, preclinical and clinical evidence requiring the need of transition from standard to selective PARPi.

PARPi have shown therapeutic efficacy across various tumors such as breast, ovarian, prostate, and pancreatic cancers. In ovarian cancers, PARPi have demonstrated significant effectiveness as first line therapy, with the therapeutic response heavily influenced due to the presence of BRCA 1/2 mutations. Olaparib has been used as combination therapy in breast cancers, while PARPi are used as post therapy in metastatic castration-resistant prostate cancer.

A significant finding is inhibition of PARP1 alone is enough to initiate the synthetic lethality in HRD tumors, while PARP2 inhibition is a major driver of hematological toxicity such as anemia, thrombocytopenia, and neutropenia, evidence has also highlighted role of potent PARP1 trapping in myelosuppression, suggesting a multifactor mechanism. The improved selective PARP1 inhibitors have insightful implications in combination therapy, that was limited due to standard PARPi, with the overlapping hematological toxicity of PARPi and platinum-based chemotherapy. Preclinical data of saruparib have shown improved antitumor efficacy without the severe side effects of hematological toxicity, however, majority of these findings are derived from preclinical models and early phase trials. Safety, long term efficacy and tolerability in large patient population remains limited. These findings collectively suggest that next-generation selective PARP1 inhibitors may be promising as a combination therapy and needed to be fully clinically validated.

Despite the advances, significant challenges remain as resistance to PARP inhibition might recur. Due to this resistance, checking BRCA status as a predictive biomarker alone is not sufficient. Thus, expanding biomarker strategies to include functional RAD51 assay, HRD scoring, and SLFN11 will help in guiding patient selection, resistance detection, and inform therapeutic strategy. Similarly, the emergence of new agents such as AZD9574, KBD111, HSK46256, and CKD-9001 have demonstrated promising results with potential applications across broader oncology settings, with potential therapeutic efficacy from HRD solid tumors into CNS malignancies.
